# Assessing the threat of *Yersinia pestis* harboring a multi-resistant IncC plasmid and the efficacy of an antibiotic targeting LpxC

**DOI:** 10.1128/aac.01497-24

**Published:** 2025-01-30

**Authors:** Nadine Lemaitre, Amélie Dewitte, Faniry Rakotomanimana, David Gooden, Eric Toone, Minoarisoa Rajerison, Pei Zhou, Florent Sebbane

**Affiliations:** 1 Univ. of Lille, CNRS, Inserm, CHU Lille, Institut Pasteur de Lille, U1019—UMR 9017—CIIL—Center for Infection and Immunity of Lille 56099 , Lille, France; 2 UR4294, Agents Infectieux, résistance et chimiothérapie (AGIR), Université de Picardie Jule Vernes, Amiens, France; 3 Plague Unit, Institut Pasteur de Madagascar 294567 , Antananarivo, Madagascar; 4 Department of Chemistry, Duke University 3065 , Durham, North Carolina, USA; 5 Department of Biochemistry, Duke University School of Medicine 12277 , Durham, North Carolina, USA; Columbia University Irving Medical Center, New York, New York, USA

**Keywords:** plague, *Yersinia pestis*, multidrug resistance, plasmid-mediated resistance, virulence, Flea, Rodents, drug targets, LpxC

## Abstract

Self-transmissible IncC plasmids rapidly spread multidrug resistance in many medically important pathogens worldwide. A large plasmid of this type (pIP1202, ~80 Kb) has been isolated in a clinical isolate of *Yersinia pestis*, the agent of plague. Here, we report that pIP1202 was highly stable in *Y. pestis-*infected mice and fleas and did not reduce *Y. pestis* virulence in these animals. Although pIP1202 inflicted a fitness cost in fleas (but not in mice) when the insects fed on blood containing a mixture of plasmid-free and plasmid-bearing strains, such a co-infection scenario has never been reported in nature, indicating that pIP1202 could persist in *Y. pestis* strains. Despite being resistant to commonly used antibiotic treatments, we show that plague caused by *Y. pestis* harboring the pIP1202 plasmid is effectively cured by LPC-233—a potent inhibitor of the essential LpxC enzyme in the lipid A biosynthetic pathway. Taken as a whole, our data highlight the alarming threat posed by *Y. pestis* harboring multidrug-resistant IncC plasmids that may persist in wild animals as a reservoir for long periods without antibiotic pressure and illuminate the impact of antibiotics with a novel mode of action against such a biothreat.

## INTRODUCTION

Multidrug resistance (MDR) diminishes the antimicrobial arsenal that protects us against medically important bacterial agents, including those responsible for some of the worst known epidemics in human history (e.g., plague and cholera) (1, 2). In the absence of effective new treatments, the spread of multidrug-resistant bacteria threatens to create a new global public health crisis with broad societal disruption and an unbearable death toll. It is therefore essential to understand the mechanisms underlying MDR, its spread, and its persistence to develop novel antibiotics that can kill multidrug-resistant pathogens.

Bacteria acquire antibiotic resistance through mutation or horizontal gene transfer (HGT) after transformation, transduction, or conjugation (3–5). Unlike point mutation, HGT provides bacteria with one or more antibiotic-resistance genes in a single block. Hence, HGT in general and conjugative transfer in particular are key mechanisms for the rapid spread of MDR genes. It is noteworthy that the range of drugs to which integrative and conjugative elements (e.g., plasmids) confer resistance has increased considerably in recent years (6, 7). When combined with the high frequency of plasmid transfer from one bacterium to another or from one bacterial species to another, these effects make conjugative plasmids one of today’s greatest threats to public health.

The large, low-copy-number incompatibility (Inc) C plasmids are among the conjugative plasmids that carry MDR genes (8–11). IncC plasmids were discovered in the late 1960s and have since been detected in a wide range of human and animal pathogens; indeed, they are now considered to be epidemic (8–14). Sequence analysis indicates that IncC plasmids evolve constantly; this evolution is in part due to the presence of multiple hotspots for the accretion of antibiotic-resistance genes. For example, IncC plasmids spread the β-lactamase-encoding genes *bla*_CMY-2_ and *bla*_NMD-1_, which pose an increasing threat to the public health system (11, 14, 15). This threat has been exacerbated by the recent detection of an IncC plasmid (pIP1202, first isolated in Madagascar in 1995) in a pure culture of a clinical strain of the plague bacillus *Yersinia pestis*; worryingly, pIP1202 confers resistance to all the antibiotics recommended by the World Health Organization for the prophylactic and curative treatments of plague (1, 16). It is also worth mentioning that the threat of antibiotic resistance is further accentuated by the fact that an MDR IncC plasmid worse than pIP1202 could be intentionally introduced into *Y. pestis* for biological warfare purposes.

*Y. pestis* is an obligate pathogen that spreads through mammalian and flea hosts. In the flea, the bacterium induces and consolidates a mass that obstructs the insect’s foregut within the 4–6 days of infection, in one of the most efficient flea vectors, the oriental rat flea, *Xenopsylla cheopis* (17, 18). The consolidated mass prevents freshly collected blood from passing into the foregut. Consequently, the drawn blood is contaminated and regurgitated into the host dermis. Ultimately, the “blocked” flea starves prior to death. The transmission of *Y. pestis* by fleas can also occur within a few hours or days after an infectious blood meal (i.e., prior to flea blockage), and transmission quickly fades away if uninfected meals are subsequently ingested (19). This is referred to as “early-phase transmission.” Regardless of the mode of transmission, *Y. pestis* regurgitated by fleas spreads from the dermis into the draining lymph node, where the active replication produces a swollen, enlarged, necrotic, and painful lymph node, the so-called bubo, which characterizes bubonic plague. Prior bubo production, the infected lymph node is overwhelmed, and the patients showing bubo die of a fatal septicemia if left untreated in 40%–60% of the cases (20, 21). At the terminal stage of infection, *Y. pestis* will have heavily colonized all the body’s organs and produced a very high bacteremia (>10^8^ colony-forming units [CFU] per milliliter) for efficient transmission to fleas (22). In up to 5% of the cases, the hematogeneous spread of *Y. pestis* produces fatal pneumonia, allowing aerosol transmission from human to human. The newly infected patient develops the so-called primary pneumonic plague, which is fatal in >90% of the cases if left untreated.

Since the carriage of plasmids usually entails a cost for the host bacterium (23), we first investigated the impact of the multidrug-resistant plasmid pIP1202 on *Y. pestis*’ life cycle, in order to determine whether the pIP1202-positive bacillus could persist in nature for a long period. Finally, with the goal of providing a valuable treatment for this public health threat, we also looked at whether the unique mode of action of a novel antibiotic targeting LpxC, a protein that catalyzes the first committed step in the LPS synthesis (24), could enable these drugs to effectively overcome pIP1202-associated resistance in a murine model of bubonic plague.

## RESULTS

### *Y. pestis* harboring pIP1202 is fully competent in rodent and fleas

**Fig 1:**
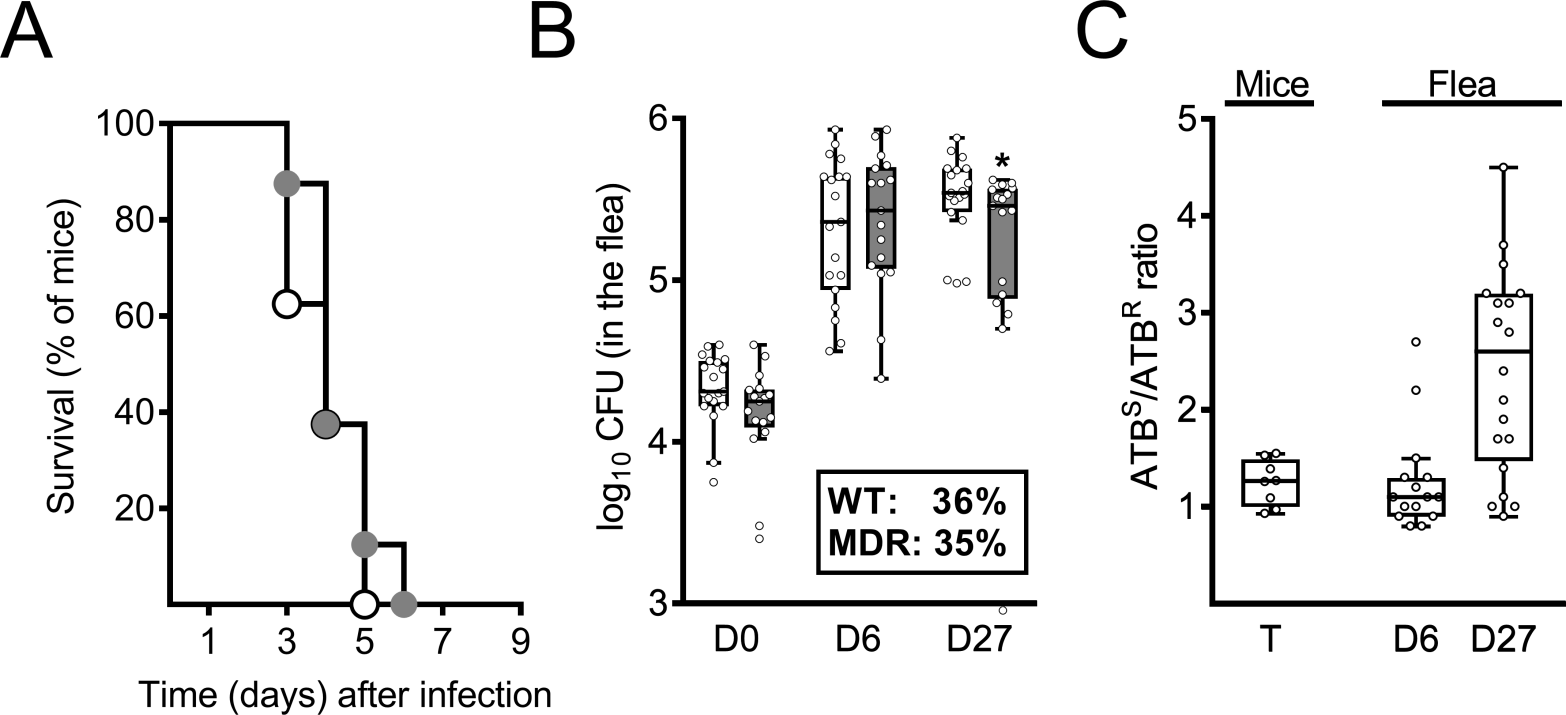
Virulence of *Y. pestis* harboring pIP1202 and its parental (WT) strain in fleas and mice, and the plasmid’s stability *in vivo*. (**A**) Survival curves of mice (*n* = 8 for each group) inoculated intradermally with ~80 CFU of the CO92 strain harboring pIP1202 (gray) or not (white). The curves did not differ significantly (*P* = 0.228 in a log-rank test). (**B**) Overlay of a Tukey style box-and-whisker plot and scatter plot showing the bacterial load in the flea on days (**D**) 0, 6, and 27 after a blood meal contaminated with 5 × 10^8^ *Y. pestis* KIM6+/mL harboring pIP1202 (gray, *n* = 17 for each time point) or not (white, *n* = 19 for each time point). The value of the data point below the X-axis (gray, **D27**) is 1. The curves did not differ significantly (*P* > 0.08 in a two-way analysis of variance). However, Sidak’s test for multiple comparisons indicated that the bacterial load in fleas infected with a multidrug-resistant strain harboring pIP1202 was significantly lower (*P = 0.0423). The inset shows the blockage rate determined using fleas (*n* = 100; equal numbers of males and females) infected with the strain harboring pIP1202 or not (WT) over a period of 4 weeks. (**C**) Overlay of a Tukey style box-and-whisker plot and scatter plot showing the ratio between the number of antibiotic-sensitive bacteria (ATB^S^) and antibiotic-resistant bacteria (ATB^R^) and isolated from (i) the spleen of mice (*n* = 8) showing the terminal stage of bubonic plague (**T**) following intradermal inoculation of ~80 CFUs of the strain harboring pIP1202 and (ii) from fleas infected with *Y. pestis* harboring pIP1202 and collected on day (**D**) 6 (*n* = 15) and D27 (*n* = 20) post-infection. Panel B shows the total number of bacteria in fleas infected with the MDR strain, whereas panel C illustrates the proportion of these bacteria that retained the MDR plasmid using the ATB^S^/ATB^R^ ratio. Additionally, panel C provides the ATB^S^/ATB^R^ ratio based on bacterial counts from the mice used to generate panel A.

Given that (i) a plasmid can be a burden for the bacterial host and (ii) the natural history of *Y. pestis* infection involves a unique flea-mammal-flea cycle (5, 25), we first determined whether plasmid pIP1202 had a negative effect in this system. To this end, we first transferred plasmid pIP1202 (from the original *Y. pestis* clinical isolate) to naive *Y. pestis* by conjugation. As expected, the recipient *Y. pestis* strain was highly resistant to several antibiotics (doxycycline, streptomycin, and chloramphenicol) but remained sensitive to gentamicin and ciprofloxacin (Table 1). It is noteworthy that we found a > 2,000-fold increase in the MIC values for doxycycline and streptomycin, both of which remain front-line therapeutics for plague in low-income countries. We next assessed the respective abilities of the pIP1202-positive and its parental strain to produce a bubonic plague in mice and a transmissible infection in fleas (i.e., flea blockage). Our results showed that the *Y. pestis* strains CO92 and JHUPRI harboring pIP1202 and their parental strains killed all mice following the intradermal inoculation of a bacterial load similar to that transmitted by a flea bite (80 CFUs) (22) (Fig. 1A; Fig. S1). In contrast to the pIP1202-positive CO92 strain, the fatal plague caused by the pIP1202-positive JHUPRI strain had a slight lag (a median survival time of 6 days vs 4 days for the WT). However, the survival curves of mice infected with JHUPRI strain harboring or not pIP1202 did not differ significantly (*P* > 0.11 in a log-rank test). We also found that *Y. pestis* strains CO92 and KIM6+ harboring pIP1202 blocked as many fleas as the corresponding WT did (52% vs 52% for CO92, and 35% vs 36% for KIM6+). Consistently, the proportion of these fleas that still contained the pIP1202-positive strain after an infected meal was 100% after 6 days and 95% after 27 days (Fig. 1B). However, although the median bacteria load recovered from fleas infected with the pIP1202-positive strain was similar to that of the parental strain at 6 days post-infection, it was statistically lower at 27 days (Fig. 1B). Nevertheless, the difference between the median bacterial counts at 27 days was very small (5.48 vs 5.54 for the pIP1202-positive and WT strain, respectively) and so may not be biologically significant. Finally, when we counted the bacteria in the spleen of mice that developed terminal bubonic plague, we found that the ratio between the number of bacteria growing on agar plates lacking antibiotics and those growing on agar plates supplemented with antibiotics was close to 1, indicating that the majority of bacteria were resistant to the antibiotics (Fig. 1C). We found a similar ratio (i.e., close to 1) when counting the bacteria collected from fleas at 6 days post-infection, and a ratio of 2.5 in bacteria collected from fleas at 4 weeks post-infection. Overall, these data suggest that (i) *Y. pestis* harboring pIP1202 is fully competent for infecting mice and producing a transmissible infection in fleas, and (ii) without antibiotic pressure, the plasmid pIP1202 is stable over the course of the infections.

**TABLE 1:** *In vitro* activity of LPC-233 and other antimicrobial agents against *Y. pestis* strains harboring pIP1202^*a*^

Strain	MIC (µg/mL) or susceptibility (S) or resistance (R) to antibiotic:					
	LPC-233	Dox	Sm	GM	CAM	CIP
KIM6+	0.06	0.5 (S)	2 (S)	0.5 (S)	4 (S)	0.03 (S)
KIM6+ pIP1202	0.06	>1,024 (R)	>2,048 (R)	1.0 (S)	>32 (R)	0.03 (S)
CO92	0.06	S	S	S	S	S
CO92 pI1202	0.06	R	R	S	R	S
JHUPRI	0.06	S	S	S	S	S
JHUPRI pI1202	0.06	R	R	S	R	S

^*a*^ Dox, doxyclyline; Sm, streptomycin; GM, gentamincin, CAM, chloramphenicol; CIP, ciprofloxacin; S, susceptible; R, resistant.

### *Y. pestis* outcompetes its sister strain harboring pIP1202 in fleas but not in mice

**Fig 2:**
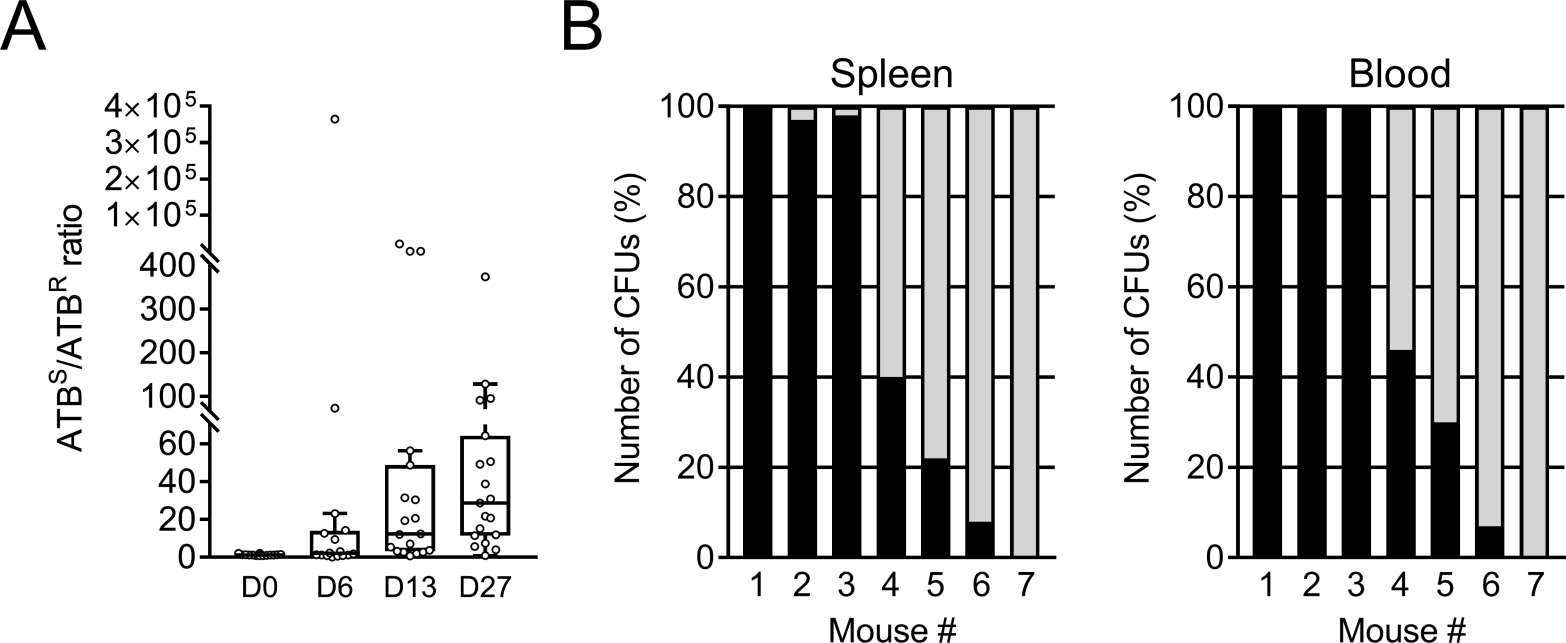
Competition experiments in fleas and mice infected with equal numbers of *Y. pestis* pIP1202 and its WT strain. (**A**) Overlay of a Tukey style box-and-whisker plot and scatter plot showing the ratio between the number of antibiotic-sensitive bacteria (ATB^S^) and antibiotic-resistant bacteria (ATB^R^) *Y. pestis* isolated from fleas (*n* = 13–19) sampled immediately (**D0**) or on days (**D**) 6, 13, and 27 after feeding on blood contaminated with a bacterial suspension comprising equal numbers (2.5 × 10^8^ CFUs) of *Y. pestis* harboring pIP1202 (multidrug-resistant) or not (WT). (**B**) The percentage of *Y. pestis* growing on an agar plate supplemented with antibiotic (black) or not (gray) and isolated from the spleen and blood of mice inoculated intradermally with an inoculum comprising a 1:1 mix (~80 CFUs) of *Y. pestis* harboring pIP1202 (multidrug-resistant) or not (WT).

Many plasmids are known to weaken the competitiveness of the bacterial host in the absence of selective pressure, unless the plasmid-bearing strain has compensatory mechanisms (23). We therefore considered evaluating the fitness cost associated with plasmid pIP1202 by co-infecting fleas and mice with equal numbers of pIP1202-positive and parental *Y. pestis* cells; this type of *in vivo* competition approach amplifies any fitness cost and thus makes it possible to detect small defects in virulence. We found that when the inoculum contained equal numbers of parental and pIP1202-positive *Y. pestis* bacteria, the parental:pIP1202 ratio was ~20 after 27 days of incubation; this provided solid evidence of a fitness cost of the pIP1202 carriage by *Y. pestis* in the flea (Fig. 2A). The same competition experiment in intradermally inoculated mice did not yield a consistent trend. After inoculation with equal numbers of parental and pIP1202-positive strains, examination of the bacterial content in the spleens and blood of individual mice showed large variations in the parental-to-pIP1202 colony ratio; this ranged from an infection dominated by the WT (in three of the seven mice) to an infection dominated by the strain harboring the pIP1202 (in three other mice) (Fig. 2B). These observations suggest that plasmid pIP1202 does not incur an obvious fitness cost in mice.

### A potent LpxC inhibitor efficiently controls multidrug-resistant *Y. pestis in vitro*

The results of our virulence experiments suggested that although *Y. pestis* harboring pIP1202 has a fitness disadvantage when the fleas are co-infected with the WT *Y. pestis* strain, it is nevertheless able to maintain a flea-rodent-flea cycle when infecting the hosts. It is noteworthy that the bacterial co-infection scenario in the flea has never been reported in nature, and if it occurs, it must be infrequent due to a restriction in bacterial dissemination from the skin to other tissues (26, 27); blood from infected animals rarely contains a mixture of pIP1202-positive and parental strains. It is also worth noting that the pIP1202-hosting strain dominates or is present in almost equal numbers to the parental strain in ~50% and 25% of fleas at days 6 and 13 post-infection, a period of time where blocked fleas are diagnosed and transmit *Y. pestis* (22). Furthermore, fleas can transmit *Y. pestis* efficiently within a few hours or days after an infectious blood meal, prior to flea blockage (19). In other words, the dynamics of transmission of *Y. pestis* by fleas suggest that fleas can transmit efficiently a strain harboring the pIP1202 plasmid before the fitness cost associated with pIP1202 showed its full effect. Therefore, *Y. pestis* carrying pIP1202 or any IncC MDR plasmid is likely to persist for an extended period of time in the absence of antibiotic selection pressure. Given the ability of *Y. pestis* to acquire various antibiotic resistance plasmids in nature (1, 28, 29), the stability of the IncC plasmid without any selective antibiotic pressure in *Y. pestis* (Fig. 1C), and the capacity of IncC plasmid to aggregate resistance genes (including to resistance to ciprofloxacin, the first line of recommendation against plague in rich countries) (14, 30, 31), there is an urgent need for novel antibiotics designed to avert a pending epidemic. Our research groups have previously reported that antibiotics targeting LpxC (an essential enzyme for the biosynthesis of the lipid A component of the outer membrane of Gram-negative bacteria) can cure bubonic plague (the most common form of the disease) in mice (32). Here, we evaluated whether LpxC inhibitors could protect against plague caused by multidrug-resistant *Y. pestis* harboring pIP1202. Like other IncC plasmids, pIP1202 harbors a large number of uncharacterized resistance genes (16), and whether they could cause resistance to LpxC inhibitors is unknown.

We took advantage of our current study to evaluate LPC-233, a new LpxC inhibitor selected from a collection of over 200 LpxC-targeting compounds (33). LPC-233 is extremely efficacious against multiple strains of *Y. pestis* (KIM6+, CO92, and JHUPRI), with MIC values of 0.06 µg/mL (Table 1; Fig. S2). These MIC values are nearly 10-fold lower than that for LPC-069, which has been shown to be efficacious in a bubonic model of plague (32). Such a dramatic reduction in the MIC probably reflects LPC-233’s potent inhibitory effect. Importantly, in contrast to several antibiotics used to treat plague, LPC-233’s efficacy is similar in both the pIP1202-positive strain and the WT strain; this highlights the potential of novel LpxC inhibitors to overcome multidrug-resistant *Y. pestis* infections (Table 1). Interestingly, tests of LPC-233 with streptomycin or doxycycline did not reveal synergy in the pIP1202-positive *Y. pestis* strains, presumably due to the extreme resistance to streptomycin and doxycycline conferred by the plasmid (Fig. S2).

### LpxC inhibitor prevents lethal multidrug-resistant *Y*. *pestis* infections in mice

Our pharmacologic experiments indicated that intraperitoneally administered LPC-233 has a longer half-life in mice than intravenously administered LPC-069 (43 min vs 10 min, respectively) (32). The blood concentration peaked at 8.0 µg/mL (130 times the MIC) after 30 min (Table S1). Furthermore, intraperitoneal administration of the highest dose level of LPC-233 tested in rodents (200 mg/kg q12h for 5 consecutive days) was not associated with any noteworthy adverse events. These data are consistent with our previous work showing the safety of LPC-233 in other animal models, including dogs (33). Hence, we next investigated the compound’s ability to protect against plague caused by *Y. pestis* strain harboring or not pIP1202 in mice. More specifically, mice were inoculated with ~80 CFUs by intradermal route (as the flea does) and then treated with a 5-day regimen with 40 mg/kg q12h intraperitoneally when *Y. pestis* is known to initiate the colonization of the draining lymph node but not the blood (i.e., 18 h post-inoculation) (20, 34). The regimen was chosen based on our previous investigation using LpxC inhibitors (i.e., LPC-058 and LPC-069) (32), and our previously published (33) and present (Table S1) pharmacokinetic and toxicity data associated with LPC-233.

**Fig 3:**
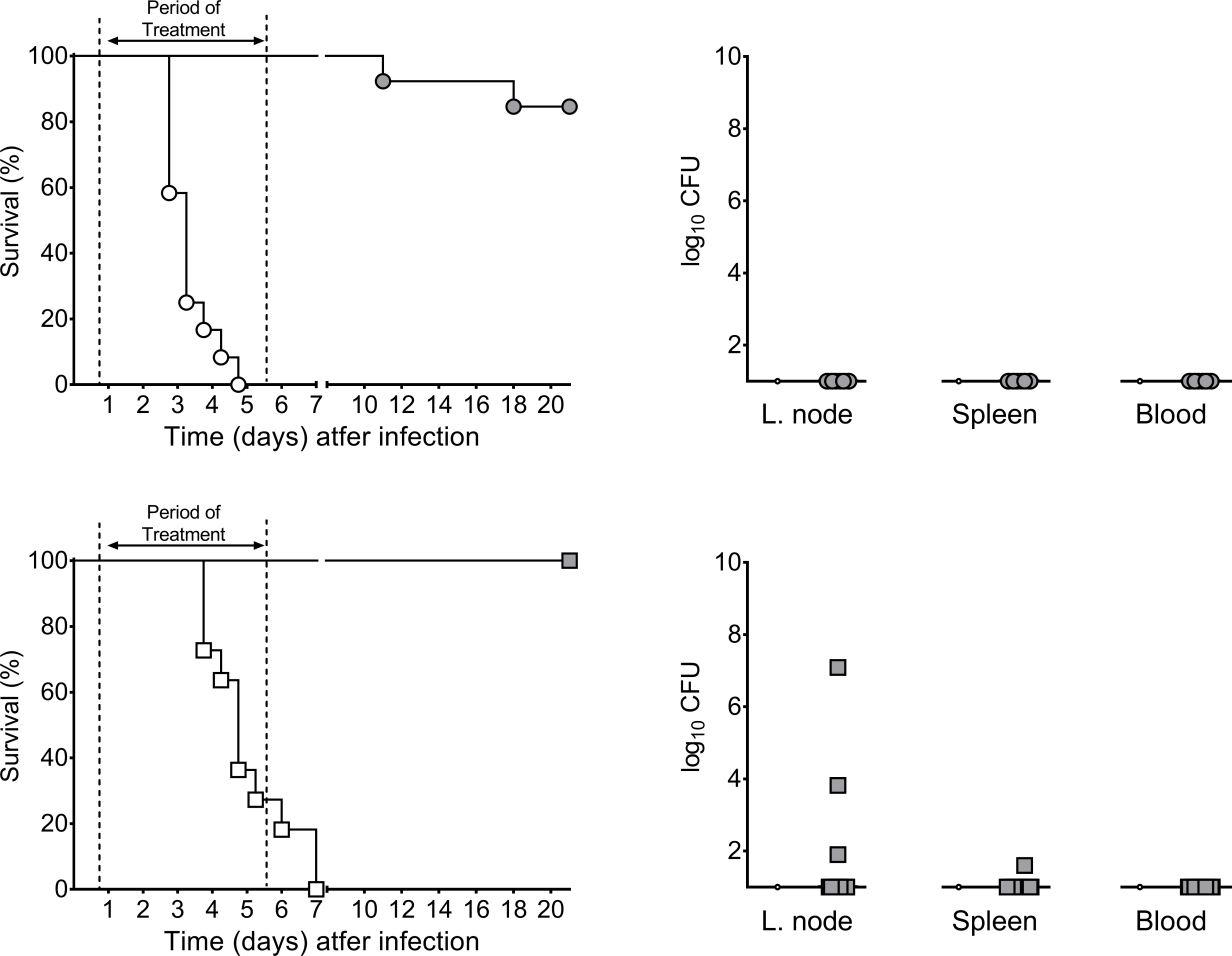
Efficacy of LPC-233 against bubonic plague caused by multidrug-resistant and non-multidrug-resistant *Y. pestis*. Eight- to 9-week-old OF-1 female mice were inoculated intradermally with ~80 CFU of *Y. pestis* CO92 harboring pIP1202 (squares) or not (circles). Eighteen hours later, mice were given a 5-day course of intraperitoneal LPC-233 (40 mg/kg q12h, gray symbols) or not (white symbols). The two curves differed significantly (*P* < 0.0001 in a log-rank test). Bacterial loads in the survivors' draining lymph nodes, spleen, and blood were determined 18 h after the end of the course of treatment. A group of 12 and 11 for untreated mice infected with the CO92 strain and the CO92 harboring pIP1202 were used, respectively. A group of 11 and 13 mice for treated mice infected with the CO92 strain and the CO92 harboring pIP1202 were used, respectively. For the enumeration of the bacteria in the survivors, 10 and 12 mice were used.

Regardless of the strain used, none of the 26 mice displayed any signs of illness during the treatment period. Two weeks after the end of the course of treatment, >80% of mice infected with the WT strain and 100% of mice infected with pIP1202-positive strain were alive, whereas none of the mice in the control groups treated with vehicle survived (Fig. 3). Bacteriologic testing indicated that all survivors infected with the WT strain lacked bacteria in their draining lymph nodes, spleen, and blood. Of the survivors infected with the pIP1202-positive strain, three had bacteria in the draining lymph node (which showed suppuration), and one also had a few bacteria in the spleen (which showed abscesses). In other words, the majority of survivors were cured of plague, and a few survivors displayed signs that are considered to be part of the recovery stage in humans (35). Taken as a whole, these data indicate that LPC-233 is a potent antibiotic against pIP1202-positive and negative *Y. pestis*.

## DISCUSSION

The fact that no IncC-bearing *Y. pestis* strains have been isolated since the pIP1202-positive strain suggests that the acquisition and propagation of IncC plasmids in *Y. pestis* is relatively uncommon. This low likelihood of acquisition is probably due to infrequent contact between *Y. pestis* and a potential bacterial donor. Indeed, *Y. pestis* is an obligate pathogen that mostly alternates between fleas and mammalian hosts. Furthermore, genetic exchange might occur primarily in the flea’s digestive tract, rather than in *Y. pestis*-colonized mammalian tissues; the latter do not usually contain other bacteria (36). Infrequent propagation of the IncC plasmid might be related to its instability or the selective disadvantage conferred on the host strain. *In vitro* and in the absence of antibiotics, pIP1202 is highly stable in *Y. pestis* after 10 days of culture (1). In this context, it is noteworthy that the IncC plasmid (pRMH760) was reportedly stable in *E. coli* for at least 110 generations *in vitro* (37). Here, we found that plasmid pIP1202 appears to be highly stable in both rodents and fleas. We also found that *Y. pestis* harboring pIP102 is fully virulent in mice, notably when co-injected 1:1 with the WT (Fig. 1A and 2B; Fig. S1). In fleas, *Y. pestis* harboring pIP102 is also fully competent for the production of a transmissible infection (i.e., block fleas) (Fig. 1B). However, *Y. pestis* harboring pIP1202 was progressively outcompeted by the WT strain in fleas that ingested a blood meal contaminated by pIP102-positive and -negative strains (Fig. 2A). Taken as a whole, the co-infection data suggest that passage through the flea (rather than through the rodent) evicts *Y. pestis* harboring pIP1202 and that this, in turn, implies that the likelihood of rodent fleas transmitting *Y. pestis* harboring pIP1202 to humans is relatively low. However, this eviction of the *Y. pestis* harboring pIP1202 in the flea might occur mainly when the flea ingests a blood meal from animals co-infected with a mixture of pIP1202-positive and -negative strains. The literature data and our present results indicate that septicemia with two or more genetically distinct clones is rare because of a bottleneck at the flea bite and a reduction in the size of the bacterial population: in other words, a clone that passes the bottleneck completely dominates the colonization of an animal and, in turn, that clone can be completely supplanted by another clone in another animal (26, 27, 38). One reason why the pIP1202 clone was isolated only once certainly mirrors the fact that (i) the reported case of human plague harboring the pIP1202 clone was an isolated case of human plague in the Besoa region of Madagascar, (ii) the region where the pIP1202 clone has been isolated in the patient was a largely underpopulated region, and (iii) there was no field survey for such clone in this particular region; that is, the chance of transmission to human and to re-isolate it from wild-life is rare or non-existing. In conclusion, a strain of *Y. pestis* carrying plasmid pIP1202 (and possibly other conjugative plasmids) might persist in rodents and their associated fleas over relatively long periods in the absence of antibiotics. Our data further suggest that wild animals (i.e., mammals and insects) are not only temporary carriers of bacteria harboring conjugative multidrug plasmids but also act as a reservoir for these plasmids and their spreading. This alarming prospect emphasizes the need to monitor emerging antibiotic resistance in wildlife.

The IncC plasmid appears to be a highly dynamic piece of DNA capable of accumulating antibiotic resistance genes over time (11, 14, 15). Together with the possible role of animals as long-term reservoirs for the IncC plasmid and its spread to the most dangerous pathogens (such as *Y. pestis*), this capacity emphasizes the need for new treatments against multidrug-resistant bacteria. It has been reported that inhibitors of LpxC are a new class of effective antibiotics against Gram-negative clinical isolates *in vitro* and *in vivo* (32, 39–43). Notably, we reported previously that the LpxC inhibitor, LPC-069, cures plague caused by an antibiotic-sensitive strain (32). Here, we investigated whether LpxC inhibition could protect against plague caused by the *Y. pestis* strain harboring pIP1202 since this very large plasmid harbors > 80 resistance genes (16), one of which could confer resistance to LpxC inhibitors. Prior to conducting experiments with our pIP1202-positive strain, we sought to discover an inhibitor better than LPC-069. Indeed, the administration regimen for LPC-069 was cumbersome; it required the intravenous injection of 200 mg of LPC-069 every 8 h. Our investigations led us to discover LPC-233. The MIC of LPC-233 for *Y. pestis* is ~10 times lower than that of LPC-069 and those reported for gentamicin and doxycycline, both of which are recommended for the treatment of plague (Table 1). Like LPC-069, LPC-233 was non-toxic and capable of curing plague in our experiments (Fig. 3). More importantly, the dose of LPC-233 used to cure plague caused by a strain harboring pIP1202 was five times less than that of LPC-069 (40 vs 200 mg/kg, respectively), and fewer injections were required (two vs three injections per day, respectively).

In conclusion, our present data suggest that multidrug-resistant IncC plasmids are a serious threat and might persist for a relatively long term in wildlife once acquired by bacteria such as *Y. pestis*, even in the absence of selective pressure from antibiotics. Crucially, we demonstrated that this threat could be countered by the administration of LpxC inhibitors. In particular, the LpxC inhibitor LPC-233 might be a valuable addition to our future therapeutic arsenal for killing bacteria-bearing IncC plasmids or other MDR plasmids.

## MATERIALS AND METHODS

### Bacterial strains and plasmid

Clinical isolates of *Y. pestis* biovars Antiqua (JHUPRI), Medievalis (KIM6+), and Orientalis (CO92 lacking or not the pYV plasmid), and the laboratory strain *Escherichia coli* DH5α harboring pIP1202 (or not) were used in this study. pIP1202 was transferred into *Y. pestis* strains by mating with *E. coli* DH5α. The *Y. pestis* recipient strains were selected on lysogeny broth (LB) agar containing triclosan (1 µg/mL) and streptomycin; *E. coli* was sensitive to triclosan and so was counter-selected. All the experiments using the fully virulent and the vaccine/avirulent strains were performed in a BSL-3 and BSL-2 facility, respectively. Experiments were conducted under the number S-2621PAS12007-3, authorizing us to work with *Y. pestis* in our dedicated facilities.

### Flea infection

The flea model has been described previously (18). Briefly, *Xenopsylla cheopis* rat fleas were artificially fed for 1 h on heparinized mouse blood containing 5 × 10^8^ bacteria/mL grown at 37°C overnight in brain heart infusion broth. For the co-infection study, the inoculum comprised the strain harboring pIP1202 and its wild-type (WT) parental strain in a 1:1 ratio (~2.5 × 10^8^ bacteria/mL of each strain). To monitor the blockage of the flea’s digestive tract (i.e., the production of a transmissible infection), cohorts of 100 fed fleas (with equal numbers of males and females) were collected and then allowed to feed twice a week for 4 weeks. Blockage (diagnosed by the presence of fresh red blood only in the flea’s foregut) was monitored immediately after each feed rather than the first after the infection. To monitor the time course of flea colonization and in the co-infection study, groups of 80 female fleas were collected after feeding on contaminated blood and were then fed twice a week. On various days post-infection, the fleas were collected at random, immediately after a feed. Each flea was triturated individually and then spread on LB agar plates containing 1 µg/mL triclosan, 10 µg/mL hemin, and (in some experiments) 50 µg/mL kanamycin. CFUs were counted after a 48 h incubation at 28°C. The ratio between the numbers of CFUs in the presence and absence of kanamycin was used to estimate the number of drug-resistant and drug-sensitive bacteria.

### Determination of bacterial virulence

Groups (*n* = 8) of 8- to 9-week-old female OF-1 mice (Charles River Laboratories, France) were inoculated intradermally with ~80 CFUs of *Y. pestis* grown overnight at 21°C, as described previously (20). For the co-infection study, the strain harboring pIP1202 and the WT were inoculated 1:1 (i.e., ~40 CFUs of each strain). The spleen was collected immediately after euthanasia of mice displaying terminal-stage plague. The organ was triturated into PBS, and serial dilutions of the lysate were spread onto blood agar plates supplemented (or not) with 50 µg/mL of kanamycin. The CFUs were counted after a 48 h incubation at 28°C. The ratio between the numbers of CFUs in the presence and absence of kanamycin was used to estimate the number of drug-resistant and drug-sensitive bacteria.

### Assessment of LPC-233’s efficacy *in vivo*

Mice infected intradermally with ~80 *Y*. *pestis* (grown in LB at 21°C without shaking) were assigned randomly to two groups (*n* = 11–13 per group). Eighteen hours after inoculation (i.e., when *Y. pestis* is known to initiate the colonization of draining lymph nodes but not the blood of most animals) (20, 34), one group was injected intraperitoneally every 12 h for 5 days with 20% hydroxypropyl-beta-cyclodextrin in saline, whereas the other group received the LpxC inhibitor LPC-233 (40 mg/kg). The 40 mg/kg was chosen based on our previous *in vitro* and *in vivo* experiments using LpxC inhibitors, including LpxC-233 (33). Survival was monitored every 12 h until the end of the 5-day course of treatment and then every 18 h for an additional 15 days. Fifteen days after the end of the course of treatment, samples of blood, inguinal and axillary lymph nodes, and spleen were collected from survivors and spread on agar blood plates for CFU counting. Tissues containing 60 CFUs or less (i.e., below the limit of detection) were considered to be sterile.

### Pharmacokinetic studies

LC-MS/MS methods were used to measure the concentration of LPC-233 in mouse plasma, as described previously (32). LPC-233 was injected intraperitoneally at a dose of 40 mg/kg, and plasma samples were collected by cardiac puncture 0, 5, 10, 20, 30, 60, 120, 240, and 480 min after the injection. Three mice were assessed at each time point.

### LpxC inhibitors

LPC-069 and LPC-233 were synthesized as described previously (32, 33). LpxC inhibitors were dissolved in dimethyl sulfoxide (for *in vitro* studies) or in saline containing hydroxypropyl-beta-cyclodextrin (Kleptose HPB; Roquette, Lille, France) (for *in vivo* studies).

### *In vitro* susceptibility to LpxC inhibitors

Minimum inhibitory concentrations (MICs) for all the *Y. pestis* strains used in the study were determined by using the broth microdilution method in 96-well plates containing Mueller-Hinton (MH) broth and resistance phenotyping in a disk diffusion assay, according to the Clinical and Laboratory Standards Institute’s guidelines (44). The MIC (defined as the lowest concentration at which no visible growth occurred) was determined 48 h after incubation of the plates at 28°C without shaking. For the time-kill curve assay, *Y. pestis* KIM6+ harboring pIP1202 was incubated at 28°C with shaking in MH broth supplemented (or not) with 0.5×, 1×, or 2× the MIC of LPC-233, 5 µg/mL of doxycycline, or 35 µg/mL of streptomycin (corresponding, respectively, to the simulated peak level obtained after single oral dose of doxycycline 200 mg and a single intramuscular injection of streptomycin 1 g) (45, 46). At various incubation time points, the samples were collected, serially diluted, and then spread onto blood agar plates to determine bacterial counts (CFU). Bactericidal activity was defined as a killing effect ≥3-log10.

## SUPPLEMENTAL MATERIAL

Supplemental material aac.01497-24-s0001.pdf Figures S1 and S2; Table S1.

ASM does not own the copyrights to Supplemental Material that may be linked to, or accessed through, an article. The authors have granted ASM a non-exclusive, world-wide license to publish the Supplemental Material files. Please contact the corresponding author directly for reuse.
