# Supplementary material for: Assessing the threat of Yersinia pestis harboring a multi-resistant IncC plasmid and the efficacy of an antibiotic targeting LpxC
Source: Antimicrob Agents Chemother. 2025 Jan 30;69(3):e01497-24. doi: 10.1128/aac.01497-24 (PMC11881579; doi:10.1128/aac.01497-24)
Supplement: Supplemental material — Figures S1 and S2; Table S1. [file aac.01497-24-s0001.pdf]

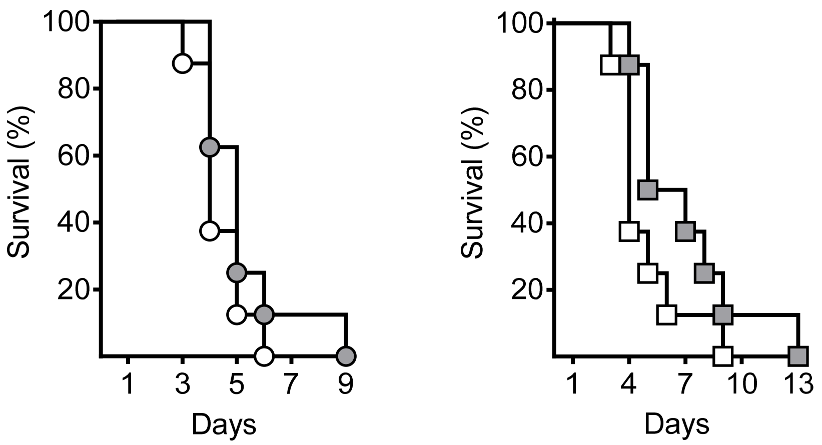

Fig.S1. Measure of the virulence of *Y. pestis* harboring pIP1202 (or not) in a mouse model of bubonic plague. Survival curves of mice (n=8) inoculated intradermally with ~80 *Y. pestis* strain CO92 (circles) or JHUPRI (squares) harboring pIP1202 (grey) or not (white). The curves did not differ significantly (P>0.1 in a log-rank test).

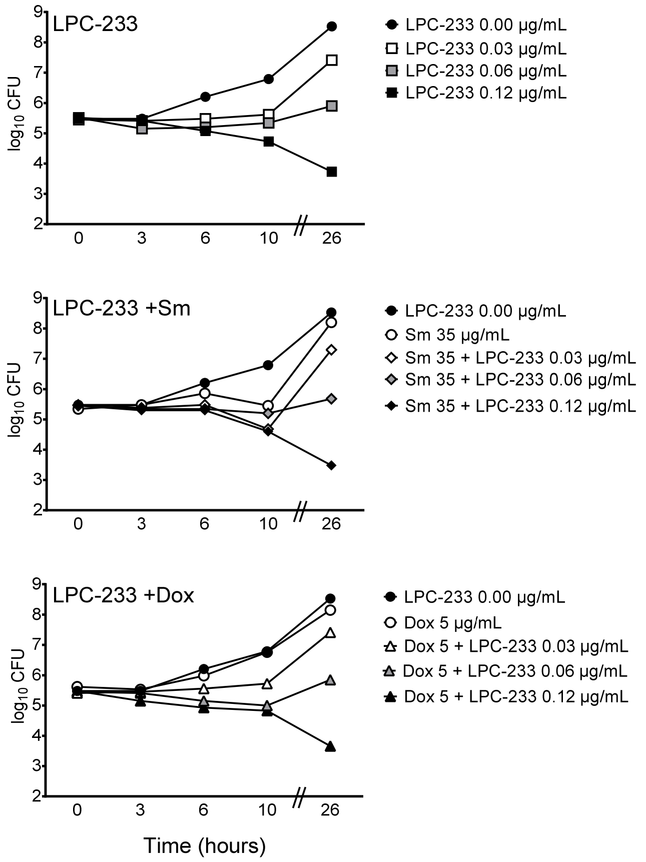

Fig. S2. Activity of LPC-233 against *Y. pestis*. Time-kill curves for *Y. pestis* KIM6+ harboring pIP1202 and grown at 28°C in Mueller-Hinton medium supplemented (or not) with 0.03 to 0.12 µg/ml of LPC-233, doxycycline (Dox; 5 µg/ml) or streptomycin (Sm; 35 µg/ml). The data are representative of two independent experiments.

Table S1. Pharmacokinetics of the LpxC inhibitor LPC-233 determined after the intraperitoneal injection of 40 mg/kg.

AUC (0-4h) (µL/h/mL)	$T_{1/2}$ (min)	C_{max} µg/mL	T_{max} (min)
9.7	43	8.0	30

$T_{1/2}$: half-life; AUC: area under the curve; C_{max}, the maxmimal concentration recorded; T_{max}; the time needed to reach C_{max}.
